# Supplementary material for: Association between oral microbiome and seven types of cancers in East Asian population: a two-sample Mendelian randomization analysis
Source: Front Mol Biosci. 2023 Nov 21;10:1327893. doi: 10.3389/fmolb.2023.1327893 (PMC10702768; doi:10.3389/fmolb.2023.1327893)
Supplement: Supplementary file 1 [file DataSheet1.ZIP › Supplementary Materials/MR plots of saliva/saliva═╝/Breast cancer/pheno.91_to_breast cancer_forest.pdf]

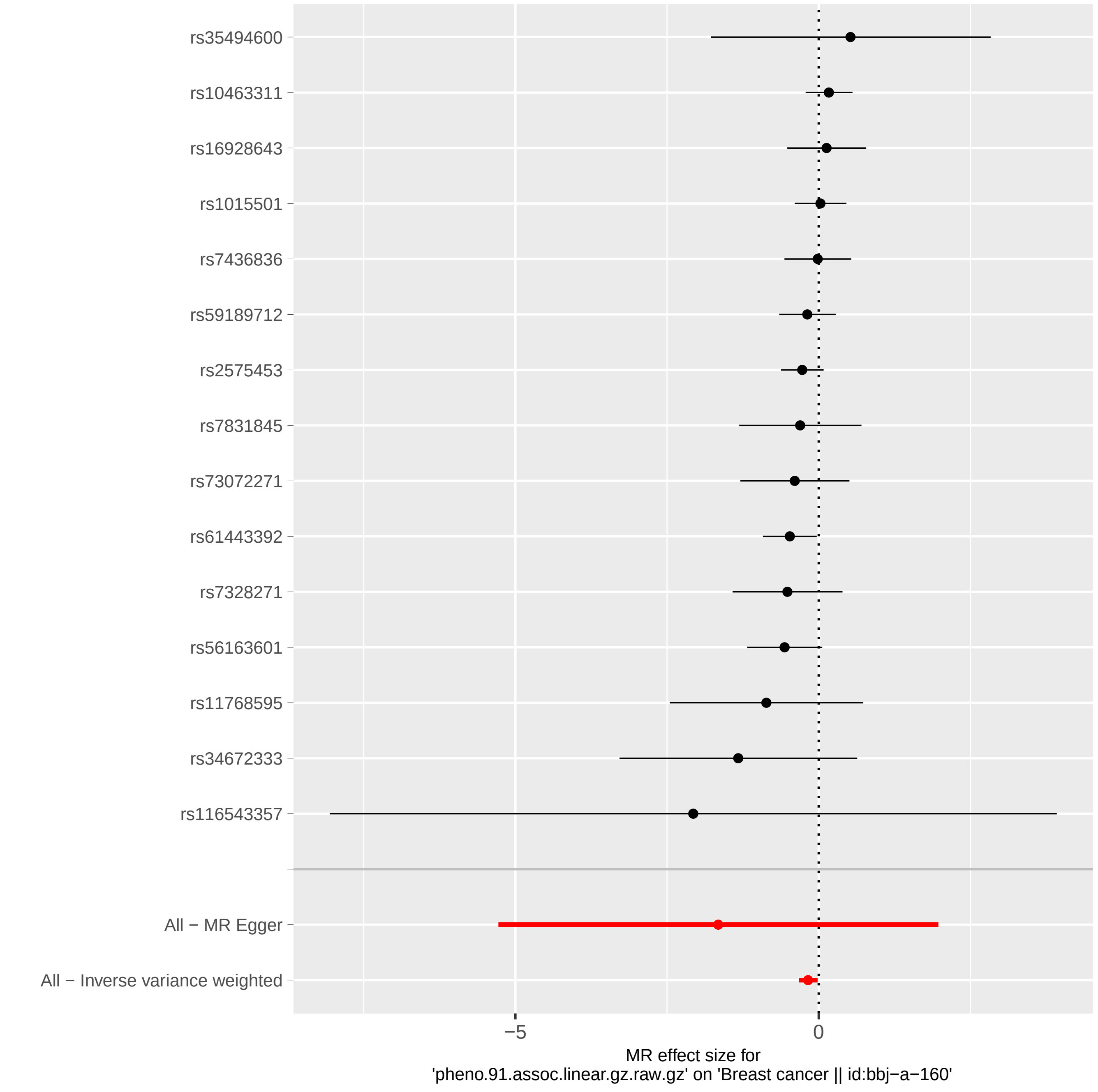

rs35494600
rs10463311
rs16928643
rs1015501
rs7436836
rs59189712
rs2575453
rs7831845
rs73072271
rs61443392
rs7328271
rs56163601
rs11768595
rs34672333
rs116543357
All − MR Egger
All − Inverse variance weighted
−5
0
MR effect size for
'pheno.91.assoc.linear.gz.raw.gz' on 'Breast cancer || id:bbj−a−160'
